# Cryobiopsy as a Salvage Technique Following Negative Flexible Forceps Biopsy of the Pleura Under Rapid On-Site Evaluation Guidance: A Prospective Study

**DOI:** 10.1155/carj/6641774

**Published:** 2025-08-12

**Authors:** Jianlong Tan, Cuihua Zhang, Bing Liu, Yun Li, Zhiguang Liu, Weidong Zhang

**Affiliations:** ^1^Department of Respiratory Medicine, Hunan Provincial People's Hospital, The First Affiliated Hospital of Hunan Normal University, Changsha 410005, Hunan, China; ^2^Clinical Medicine Research Center for Respiratory Rehabilitation in Hunan Province, Changsha 410005, Hunan, China

**Keywords:** cryobiopsy, diagnostic yield, flexible forceps biopsy, pleuroscopy, rapid on-site evaluation

## Abstract

A diffusely thickened or hard pleura is a special type of macroscopic appearance associated with benign or malignant conditions. Medical thoracoscopy (MT) is the gold standard for pleural pathology, but its diagnostic yield is imperfect. Although cryobiopsy may provide greater and deeper tissue, its impact on the diagnostic yield remains uncertain, and safety concerns persist. We evaluated the efficacy and safety of cryoprobe biopsy as a salvage technique following negative or inconclusive flexible forceps biopsy during MT under the guidance of rapid on-site evaluation (ROSE). This prospective study enrolled 280 patients with undiagnosed exudative pleural effusion who underwent MT. After the initial flexible forceps biopsy and ROSE, 37 patients with negative ROSE results underwent cryoprobe biopsy. A total of 37 (21 males and 16 females) patients, aged 56.43 ± 16.09 (range: 22–78) years with negative ROSE results, underwent cryoprobe biopsy. CB established a definitive histopathological diagnosis in 33/37 (89.2%) patients, which was significantly higher than that achieved with FFB, i.e., 21/37 (56.8%; *p*=0.002). CB resulted in significantly larger pleural specimens (9.86 ± 2.69 mm) in comparison to FFB (2.89 ± 1.15 mm, 95% confidence interval [CI]: 6.01–7.93; *p* < 0.001). Furthermore, CB was faster than FFB (median durations of 15 and 31 min, respectively; *p* < 0.001). CB had improved tissue quality for CGP testing in 20 NSCLC patients compared to FFB (18/20 versus 8/15, *p*=0.036). No significant complications were noted. Cryoprobe biopsy is a safe and effective salvage technique for patients with undiagnosed pleural effusion who show negative results on flexible forceps biopsy during MT. It provides larger, higher-quality specimens with a higher positivity rate, potentially avoiding the need for repeat procedures and facilitating timely diagnosis and treatment.

## 1. Introduction

Pleural effusion, the abnormal accumulation of fluid within the pleural cavity, is a frequently encountered clinical problem with diverse underlying causes, ranging from infections to malignancies. Accurate and timely diagnosis of pleural effusion is critical for determining appropriate treatment and improving patient outcomes [1]. Despite advances in diagnostic imaging and laboratory analysis, a significant number of pleural effusions remain undiagnosed after initial noninvasive evaluations. This diagnostic challenge often necessitates invasive procedures to obtain pleural tissues for histopathological examination. Thoracentesis, accompanied by pleural fluid analysis, percutaneous needle biopsy of the pleura, and bronchoscopy, facilitates the diagnosis of the underlying causes of pleural effusions in a significant number of instances. However, these techniques do not provide a conclusive diagnosis in approximately 37.5% of cases [2, 3].

Medical thoracoscopy (MT) has become a standard diagnostic tool for pleural effusion, offering direct visualization of the pleural surface and the ability to perform biopsy. However, the diagnostic yield of MT with pleural biopsy using conventional forceps may vary from 66.7% to 91%, depending on the underlying cause of effusion [4, 5]. Rapid on-site evaluation (ROSE) is a rapid cytomorphological diagnostic procedure that is immediately evaluated by a cytopathologist to assess the adequacy and accuracy of the material obtained during biopsy [6]. ROSE during MT provides immediate assessment of tissue samples by a cytopathologist or histopathologist, indicating the need for additional biopsies or modifications to the procedure in real time. ROSE has been shown to enhance the diagnostic accuracy and efficiency of thoracoscopic biopsies, potentially reducing the need for repeated procedures and expediting treatment decisions.

Flexible forceps biopsy (FFB), a commonly used method during MT, may not always provide sufficient tissue for diagnosis, especially in cases in which the pleura appears diffusely thickened, nodular, or hard. The limitations of this technique can lead to inconclusive results and the need for further invasive interventions. Cryobiopsy (CB) offers a promising alternative in scenarios where FFB is nondiagnostic. CB has been characterized as a safe biopsy method with a substantial diagnostic yield, initially applied in the diagnosis of endobronchial tumors [7], and more recently extended to the investigation of interstitial lung diseases [8, 9] and peripheral lung diseases [10]. As described earlier, the samples obtained by CB from the parietal pleura are significantly larger and of better quality with fewer artifacts than those obtained via FFB [11]. However, an emerging body of evidence indicates that CB does not significantly increase the diagnostic yield compared to FFB under thoracoscopic visualization [12, 13]. In contrast, Nakai [14] and colleagues discovered that CB yielded accurate diagnoses of malignant pleural mesothelioma (MPM) in all instances, whereas conventional biopsy methods only provided a diagnosis in one case. This suggests that CB is a viable alternative when conventional biopsy results are negative.

This study aims to assess the efficacy and safety of cryoprobe biopsy as a salvage technique following negative or inconclusive FFB during MT under ROSE guidance. The primary objectives are to compare specimen size, quality, positive diagnostic rate, and safety between the two biopsy methods. We hypothesize that cryoprobe biopsy, supported by ROSE, will yield larger, higher-quality specimens with a higher positive rate, thereby enhancing the diagnostic process and potentially avoiding the need for repeat invasive procedures.

## 2. Materials and Methods

### 2.1. Study Population

**Figure 1:**
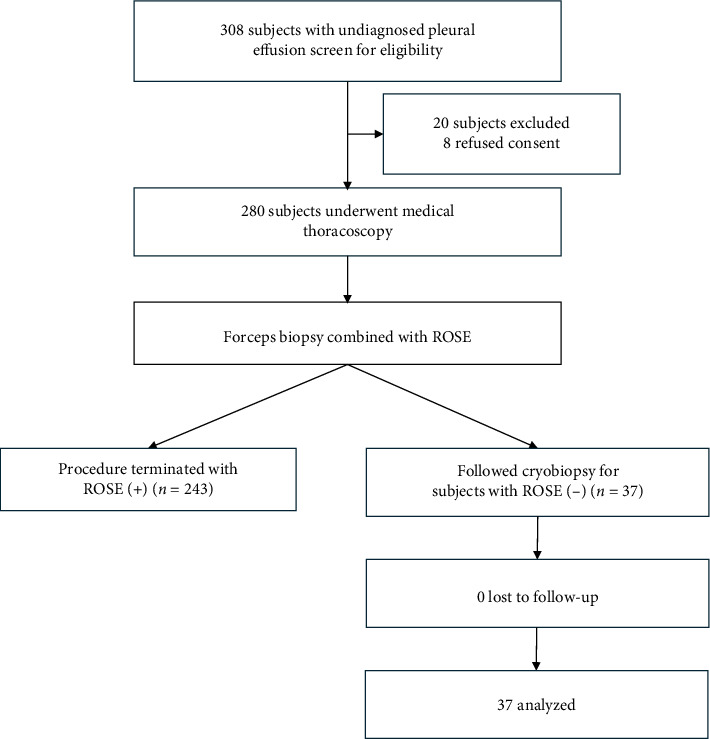
Illustration of the diagnosis flow of participants.

A total of 308 consecutive patients presenting with exudative pleural effusion of undetermined etiology were admitted to Hunan Provincial People's Hospital between January 2019 and March 2022. After a comprehensive evaluation of study eligibility, 280 patients underwent MT. The criteria for inclusion in the study were as follows: (1) age > 18 years and (2) exudative pleural effusion of unknown etiology, as determined by minimally invasive diagnostic methods. Exclusion criteria were as follows: (1) patients with absolute contraindications to MT [15, 16] and (2) individuals who did not provide written informed consent. The demographic details of the participants are documented. This prospective study was approved by the Ethics Committee of Hunan Provincial People's Hospital (no. 201873). Written informed consent was obtained from all the patients. The research protocol is illustrated in Figure 1.

### 2.2. Procedure and Equipment

All pleuroscopic examinations were conducted by an experienced operator and a trained assistant. We followed the standard British Thoracic Society guidelines for this procedure [17]. Before the initiation of the procedure, chest X-ray examination (chest CT was performed if necessary) was performed to establish the optimal entry point after artificial pneumothorax. Pethidine hydrochloride was routinely used, and local anesthesia was induced with 2% lidocaine, as described elsewhere [18]. A 1-cm incision was made along the midaxillary line, situated between the fourth and seventh intercostal spaces of the thoracic wall. Subsequently, an 8 mm flexible trocar was introduced to facilitate access to the pleural cavity, which was then exposed to atmospheric pressure. Semirigid thoracoscopy was performed using LTF-240 (Olympus, Japan). Following entry into the pleural space, pleural fluid was drained initially to explore the pleural cavity. The accessible pleural space was entirely visualized, and visual findings on macroscopic appearance were recorded. Then, forceps biopsy was performed with forceps (VDK-FB-18–105-O-O-B1, Vedkang, China) to collect multiple [19, 20] biopsy samples from either sites, deemed suspicious, present on the parietal pleura of the chest wall or diaphragm. This forceps is a flat jaw without a needle, and the “pinch-and-peel” technique was used for performing the FFB [17]. Pleural tissue was rapidly smeared on a glass slide, stained with Diff-Quik, and preliminarily diagnosed at the bedside by two cytopathologists.

According to the Guidelines of the Papanicolaou Society of Cytopathology [21], ROSE interpretation was categorized as follows: (1) Nondiagnostic specimens (e.g., no cellular or reactive mesothelial material); (2) specific benign lesions (e.g., ROSE specimens revealed granulomas, fungal, or mycobacterial); (3) atypical cells present, probably benign (e.g., the epithelial or mesenchymal component is present with nuclear atypia); (4) atypical, suspicious for malignancy (e.g., some abnormal cells are found, which have a significant risk of representing a malignant neoplasm); and (5) malignancy (e.g., cytological features indicative of malignancy are presented, allowing for precise classification of cytological subtypes). If the specimen is classified as 2 or 5, it signifies that sample collection is adequate and suitable; thus, the biopsy procedure may be terminated. However, if the assessment result is 1 or if the sampling outcome is 3 or 4, the physician determines that these findings are not consistent with the clinical presentation, pleural fluid analyses, and cytological examination of the pleural fluid, then a frozen pleural biopsy is indicated.

Subsequently, a 1.8-mm flexible cryoprobe (Kooland, China) was used for CB with cryogen (carbon dioxide) from the same pleural sites as for conventional biopsies. The specific process for pleural CB is as follows: According to an expert statement for transbronchial lung CB [22], the pressure of the frozen air source was 55–65 bar, and the cooling time was between 5 and 7 s. After ice crystals were formed at the front end, the thoracoscope and cryoprobe with the biopsied pleural tissue attached were removed simultaneously. Biopsy samples were thawed in saline solution. After the procedure, a 10Fr drainage tube (Redax, China) was placed to monitor and drain pleural fluid. A follow-up chest radiograph was prescribed 24 h after the biopsies.

The size and quality of all specimens were subsequently surveyed based on a previously published study [11]. A definitive diagnosis was established based on the histopathological, clinical, and microbiological data. The tissues were examined by two experienced pathologists who were blinded to the study design after H&E or IHC staining. The diagnostic criteria included: (a) benign lesions demonstrating characteristic pathological features with corroborating mycobacterial/bacterial culture results or (b) malignant lesions exhibiting definitive pathological characteristics. In non–small cell lung cancer cases, the quality of the sample was evaluated according to current guidelines. Slides containing more than 20% tumor cells qualified for cancer genetic panel (CGP) testing [23]. All patients without a definitive etiological diagnosis were followed for a minimum of 12 months. Nonspecific pleuritis (NSP) was determined as the final diagnosis in patients whose histopathological findings were consistent with this condition, and no evidence of malignancy or alternative diagnoses emerged from the conclusion of the follow-up period.

### 2.3. Complications

Complications associated with both techniques were also recorded. Biopsy sites were evaluated for bleeding prior to subsequent biopsies, as described elsewhere [11]: (1) no or slight bleeding, that is, bleeding volume less than 5 mL and no medication needed; (2) mild bleeding, that is, bleeding volume between 5 and 10 mL, with required local injection of frozen saline and vasoactive drugs (epinephrine: 1:10,000); (3) moderate bleeding, that is, bleeding volume > 10 mL, with required electric cauterization and argon plasma intervention; and (4) severe bleeding, requiring surgical intervention.

### 2.4. Statistical Analyses

SPSS 26 (SPSS Inc., USA) was used for data analysis. Continuous normally distributed variables were presented as mean ± standard deviation (mean ± SD) and compared using Student's *t*-test; variables with a non-normal distribution were presented as median and interquartile range and compared using the rank sum test. Categorical variables are presented as percentages and frequencies, and differences were examined using the chi-squared test. The estimated difference was calculated using the 95% confidence interval (CI). Two-tailed *p* < 0.05 was deemed to be statistically significant.

## 3. Results

### 3.1. Features of the Patients and Pleural Effusions

**Figure 2:**
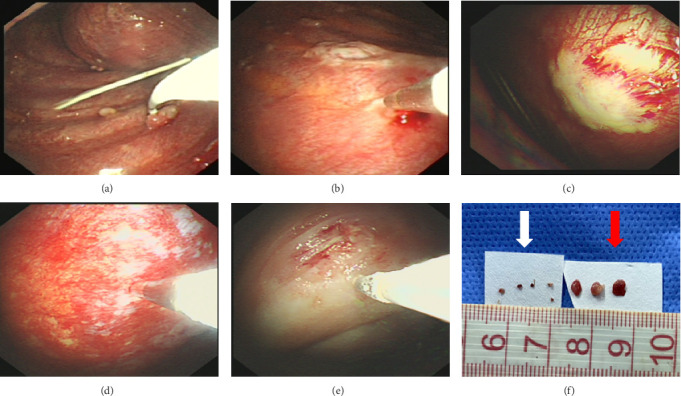
Different macroscopic appearances for pleural lesions. (a–c) Solid nodules with hard surface were detected during thoracoscopy; (d) fibrous pleura with smooth surface; (e) diffused thickened pleura was revealed by thoracoscopy; (f) compared to inadequate sampling with flexible forceps (white arrow), cryoprobe sampling was much easier from densely fibrotic pleura (red arrow).

Between January 2019 and March 2022, 280 patients with undiagnosed pleural effusion underwent thoracoscopy. Ultimately, 37 patients underwent CB following FFB because of negative ROSE outcomes. Demographic characteristics, comorbidities, and laboratory results of the pleural fluid and thoracoscopic findings are provided in Table 1. The average age of all patients was 56.4 years, and 21 of them were men. Thoracoscopic examination revealed a diffuse thickened pleura (6/37, 16.2%), solid nodules with a hard surface (7/37, 18.9%), or patchy thickened pleura with a smooth surface (24/37, 64.9%) (Figures 2(a), 2(b), 2(c), 2(d), and 2(e)). Detailed experimental datasets are provided in the Supporting Information (available here).

### 3.2. Diagnostic Yield and Sample Quality

As shown in Table 2, according to the final diagnosis, 32 subjects had malignant effusions and two had tuberculous pleuritis. In 20 subjects, the CB and FFB samples yielded the same pathological diagnosis. In another 13 cases, where samples of FFB failed to provide a definitive diagnosis, a pathological diagnosis was ultimately obtained through CB specimens. However, one subject initially classified as having NSP based on the CB specimen was eventually diagnosed with LUAD based on the FFB specimen. Six additional patients were diagnosed with NSP. Two additional patients whose pleural effusions were reduced after antitumor treatment were identified using bronchoscopy. Ultimately, pleural effusion in one patient was diagnosed as tuberculosis after empirical antituberculosis treatment.

**Figure 3:**
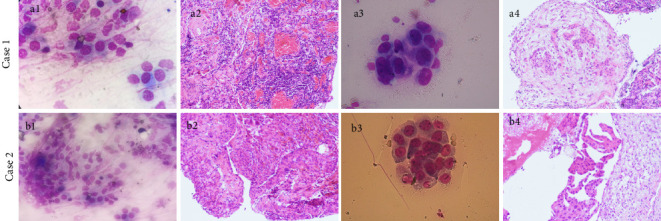
ROSE and histological comparison of biopsy techniques for 2 cases. In case 1: metastatic urothelial carcinoma of the bladder. (a1) ROSE of touch imprints of flexible forceps biopsies interpreted as mesothelial cells (Diff-Quik stain, × 100); (a2) NSP, flexible forceps biopsies shows mesothelial hyperplasia with chronic inflammatory cells (H&E stain, × 200); (a3) ROSE for cryobiopsy samples shows cancer cells with enlarged, crowded abundant cytoplasm (Diff-Quik stain, × 100); (a4) cryobiopsy reveals cancerous cells in the glandular cavity (H&E stain, × 200); In Case 2: lung adenocarcinoma. (b1, b2) ROSE of flexible forceps samples shows mesothelial cells (Diff-Quik stain, × 100) while H&E (× 200) stain was also negative. (b3) ROSE of cryobiopsy shows cancer cells arranged in clumps; (b4) the tumor cells are arranged in a papillary manner, the tumor cells are large, and the nucleoli are obvious (H&E stain, × 200).

In this study, the diagnostic yield of FFB was 56.8% among 37 patients with negative ROSE results (Figure 3). In contrast, CB provided a definitive histopathological diagnosis in 33 out of the 37 cases (89.2%), a rate that was significantly higher than that achieved with FFB (95% CI: 0.047–0.541; *p*=0.002), as shown in Table 3. The average size of the tissue sample obtained with CB was 9.86 ± 2.69 mm (range: 6–15 mm), versus 2.89 ± 1.15 mm (range: 1–6 mm) for the FFB group (Figure 2(f)). CB resulted in a significantly larger pleural specimen than FFB (95% CI: 6.01–7.93; *p* < 0.001) (Figure 2(f)). However, the duration of the CB procedure was shorter than that of FFB (median durations of 15 min and 31 min, respectively; *p* < 0.001). Furthermore, CB samples tended to contain more tumor cells on each slide for LUAD and LUSC samples before CGP testing. In the FFB group, six biopsy forceps specimens showed lung cancer cells but were insufficient for CGP testing. In the CB group, 18/20 specimens (90.0%) qualified for CGP testing (*p*=0.036) (Table 3).

### 3.3. Complications

However, complications are rare. All procedures were well-tolerated by the patients. Mild bleeding was observed in both groups (7/37 cases after CB and 5/37 after FFB, *p*=0.528). No moderate or severe bleeding occurred during the procedure or during follow-up. In addition, we documented the incidence of chest pain during the CB procedure. Only one patient experienced chest pain that necessitated local administration of lidocaine, whereas three patients reported mild chest pain that did not require any specific intervention. Furthermore, no other complications were detected postprocedure, including empyema, pneumomediastinum, pneumothorax, wound infection, and prolonged fistula (Table 3).

## 4. Discussion

In this study, we investigated the safety and efficacy of cryoprobe biopsy as a salvage technique in patients with undiagnosed pleural effusion who initially underwent FFB during thoracoscopy. Our findings suggest that cryoprobe biopsy can be a valuable alternative when the initial biopsy yields negative results, particularly in patients with diffuse thickening and nodular and hard pleura on gross appearance.

Our study demonstrated that cryoprobe biopsy specimens are larger and of better quality than FFB specimens. Tousheed [25] used a cryoprobe with a diameter of 2.4 mm and a longer freezing time, obtaining tissue samples that were significantly larger (13.2 ± 6.6 mm) than those obtained by forceps biopsy (6.6 ± 3.3 mm). Concurrently, Chen obtained significantly larger CB samples using a flexible cryoprobe with a diameter of 1.9 mm and a length of 900 mm than those obtained by FFB [11]. Despite the variations in materials and methodologies employed across individual investigations, such as cryoprobe size and freezing time, the capacity of CB to produce relatively large tissue samples is inherently evident [11, 14, 26]. The adherent cryoprobe can tear off large pieces of tissue and result in fewer instances of crush artifacts compared to forceps biopsy [12, 13]. This may be of particular importance in the diagnosis of malignant PE, as it facilitates detailed morphological, immunohistochemical, and molecular studies, as well as differentiating metastatic PE from different organs or hematological effusion from pleural mesothelioma. In this study, IHC was more easily performed with CB-based samples than FFB-based specimens in the present study (30/37 versus 18/37, *p*=0.007). CB yielded more samples with excellent tissue quality for CGP testing in 20 NSCLC patients than FFB (18/20 versus 8/15, *p*=0.036; Figure 2).

Although the routine incorporation of CB into flex-rigid pleuroscopy necessitates additional investigation [27, 28], this technique seems to enhance diagnostic capabilities, particularly in patients who exhibit negative ROSE and present with thoracoscopically apparent diffuse pleural thickening, solid nodules with a hard surface, and patchy pleural thickening with a smooth texture. Nakai et al. reported a higher diagnostic yield for samples obtained with CB (5/5) in comparison with flexible forceps (1/5) in cases with MPM, where pleuroscopy detected a thickened pleura in all patients [14]. Two studies reported a high diagnostic yield for CB in patients where FFB was negative when the macroscopic appearance showed a thickened sclerotic pleura [29, 30]. Pleuroscopic CB, which utilizes a cryoprobe, offers several advantages over the traditional biopsy methods. Primarily, CB facilitates the acquisition of large, full-thickness pleural specimens, irrespective of the hardness of pleural thickening. Pleura, which is hard and thickened, frequently results in false-negative diagnoses using FFB owing to its limited jaw opening [12, 13]. CB only requires attachment perpendicular to the pleura, and the frozen tissue specimen was obtained by pulling regardless of the hardness of the pleural thickening. As shown in Figure 2, we successfully obtained adequate tissue samples from the hard and thickened pleura, including adipose tissue. Second, the flexible nature of the forceps reduces the mechanical strength. As a result, the “pinch-and-pull” biopsy method in MT has reduced reliability, particularly in cases of thick, smooth pleural plaques. This disadvantage can be magnified if flexible forceps cannot be placed perpendicular to the intended sampling area. However, cryoprobes offer an overt advantage, as biopsies may be obtained even when the probe is placed tangentially over the pleura [13]. The results of this study indicate that cryoprobe biopsy is a more effective method for diagnosing pleural disease in certain subsets of patients. This higher positivity rate can potentially reduce the need for additional invasive procedures and facilitate timely treatment.

ROSE during MT provides immediate assessment of tissue samples by a cytopathologist or histopathologist, indicating the need for additional biopsies or modifications to the procedure in real time [31]. ROSE has been reported to be more accurate than thoracoscopists' impression of macroscopic appearance to improve the diagnostic yield of biopsy for MT with good intermodality agreement between ROSE and histopathology [32]. However, we observed a significant discrepancy between ROSE and final histopathology when the gross thoracoscopic appearance included pleural thickening and a hard texture. This poor agreement suggests that conventional biopsy forceps may not be adequate for sampling in such cases, potentially leading to false-negative ROSE results owing to severe specimen compression and superficial tissue acquisition.

In addition, this study demonstrated that using a cryotechnique during MT is safe. A higher incidence of mild bleeding was observed during the CB. However, bleeding was self-contained, and no further interventions were needed, corroborating previous reports investigating pleuroscopic CB [26, 33, 34]. No other complications were observed in this study. These findings are consistent with those of previous research [35], which demonstrated the safety of CB during flexi-rigid pleuroscopy.

Several limitations of this study should be considered when interpreting our results. First, the sample size was relatively small, which may limit the generalizability of the findings. Second, the study was conducted at a single center, and multicenter studies are needed to validate our results. Finally, the experience and skill of the operators performing the biopsies may have influenced the outcomes. Future studies should focus on larger multicenter trials to further validate the efficacy and safety of cryoprobe biopsy as a salvage technique. In addition, research on the optimal timing and indications for cryoprobe biopsy in patients with undiagnosed pleural effusions would be beneficial.

## 5. Conclusion

In conclusion, our study demonstrated that cryoprobe biopsy is a safe and effective salvage technique for patients with undiagnosed pleural effusion who have negative results from FFB during MT. Cryoprobe biopsy can provide larger, higher-quality specimens with a higher positivity rate, potentially avoiding the need for repeat procedures and facilitating timely diagnosis and treatment.

## Figures and Tables

**Table 1:** Clinical characteristics and physical/chemical data of pleural effusions.

Variables	*N*% or median (range)
Age, yrs, mean ± SD	56.4 ± 16.1

Males, *n* (%)	21 (56.7)

*Comorbidity, n (%)*	
None	21 (56.8)
Diabetes	3 (8.1)
Malignancy	3 (8.1)
Pulmonary embolism	1 (2.7)
Chronic kidney disease	1 (2.7)
Chronic hepatic disease	1 (2.7)
Pneumoconiosis	1 (2.7)
COPD	6 (16.2)

*Side of effusion, n (%)*	
Right	14 (37.8)
Left	16 (43.2)
Bilateral	7 (18.9)

*Size of effusion, n (%)*	
Small	4 (10.8)
Moderate	24 (64.9)
Large	9 (24.3)

*Results of pleural fluid biochemical analysis*	
LDH (IQR) (U/L)	305.57 (207.59–609.95)
Glu (IQR) (mmol/L)	7.08 (4.82–8.78)
Total protein (IQR) (g/L)	41.60 (33.19–50.30)
ADA (IQR) (U/L)	9.35 (6.50–14.35)

*Thoracoscopic findings, n (%)*	
Diffused thicken pleura	6 (16.2)
Solid nodules with hard surface	7 (18.9)
Patchy thickened pleura with smooth surface	24 (64.9)

*Note:* The size of a given pleural effusion was classified as small, moderate, or large based on CT imaging according to the methods described by Moy and colleagues [24]. LDH, lactate dehydrogenase; Glu, glucose; ADA, adenine deaminase.

Abbreviation: COPD, chronic obstructive pulmonary disease.

**Table 2:** Histopathological diagnosis by CB and FFB and final diagnoses of the study subjects.

Histological diagnosis based on CB	Histological diagnosis based on FFB	Final diagnosis
LUAD [17]	LUAD [12]	LUAD [17]
	Inadequate tissue [3]	
	NSP [2]	

Breast cancer [6]	Breast cancer [3]	Breast cancer [6]
	Inadequate tissue [2]	
	NSP [1]	

LUSC [3]	LUSC [2]	LUSC [3]
	Inadequate tissue [1]	

Mesothelioma [2]	NSP [2]	Mesothelioma [2]

Urothelial carcinoma of the bladder [1]	NSP [1]	Urothelial carcinoma of the bladder [1]

Granuloma with coagulative necrosis [1]	NSP [1]	Tuberculous pleuritic [1]

NSP [7]	LUAD [1] NSP [6]	LUAD [1] LUAD [2] (by TBLB) tuberculous pleuritic (1 empiric antituberculosis) NSP [3]

*Note:* LUAD, lung adenocarcinoma; LUSC, lung squamous cell carcinoma; NSP, nonspeciAc pleuritis; TBLB, transbronchial lung biopsy.

**Table 3:** Diagnostic yields, sizes, sample quality, and complications.

	CB	FFB	*p* value
Diagnostic yield	33/37 (89.2%)	21/37 (56.8%)	0.002
Number for samples (IQR)	6 (6–7)	6 (4–7)	< 0.001
Duration of the biopsy procedure (min)	15	31	< 0.001
Length, mm, mean ± SD	9.86 ± 2.69	2.89 ± 1.15	< 0.001
For IHC staining, *n* (%)	30/37 (81.1%)	18/37 (58.1%)	0.007
For CGP testing, *n* (%)	18/20 (90.0%)	8/15 (53.3%)	0.036
Bleeding, *n* (%)			
No	30 (81.08)	32 (86.49)	0.528
Mild	7 (18.92)	5 (13.51)	
Moderate/severe	0	0	

*Note:* CGP, cancer genetic panel testing; IHC, immunohistochemistry.

## Data Availability

The data that support the findings of this study are available from the corresponding author upon reasonable request.

## Nomenclature

MT: Medical thoracoscopy
CB: Cryobiopsy
FFB: Flexible forceps biopsy
TP: Total protein
LDH: Lactate dehydrogenase
ADA: Adenosine deaminase
Glu: Glucose
IHC: Immunohistochemistry
CGP: Cancer genetic panel
NSCLC: Non–small cell lung cancer
ROSE: Rapid on-site evaluation

## Supporting Information

The supporting information associated with this article includes the original datasets supporting the research findings. These data are available to facilitate reproducibility and further analysis. The supporting information can be accessed via the journal's online platform.
